# Role of Exercise in the Management of Diabetes Mellitus: the Global Scenario

**DOI:** 10.1371/journal.pone.0080436

**Published:** 2013-11-13

**Authors:** Zar Chi Thent, Srijit Das, Leonard Joseph Henry

**Affiliations:** 1 Department of Anatomy, Faculty of Medicine, Universiti Kebangsaan Malaysia, Kuala Lumpur, Malaysia; 2 Physiotherapy Programme, Faculty of Health Sciences, Universiti Kebangsaan Malaysia, Kuala Lumpur, Malaysia; Iran University of Medical Sciences, Iran (Islamic Republic Of)

## Abstract

**Background:**

Exercise training programs have emerged as a useful therapeutic regimen for the management of type 2 diabetes mellitus (T2DM). Majority of the Western studies highlighted the effective role of exercise in T2DM. Therefore, the main aim was to focus on the extent, type of exercise and its clinical significance in T2DM in order to educate the clinicians from developing countries, especially in Asians.

**Methods:**

Pubmed, Science Direct, Scopus, ISI Web of Knowledge and Google scholar were searched using the terms “type 2 diabetes mellitus,” “type 2 DM,” “exercise,” and/or “physical activity,” and “type 2 diabetes mellitus with exercise.” Only clinical or human studies published in English language between 2000 and 2012 were included. Certain criteria were assigned to achieve appropriate results.

**Results:**

Twenty five studies met the selected criteria. The majority of the studies were randomized controlled trial study design (65%). Most of the aerobic exercise based studies showed a beneficial effect in T2DM. Resistance exercise also proved to have positive effect on T2DM patients. Minimal studies related to other types of exercises such as yoga classes, joba riding and endurance-type exercise were found. On the other hand, United States of America (USA) showed strong interest of exercise management towards T2DM.

**Conclusion:**

Aerobic exercise is more common in clinical practice compared to resistance exercise in managing T2DM. Treatment of T2DM with exercise training showed promising role in USA. A large number of researches are mandatory in the developing countries for incorporating exercise in the effective management of T2DM.

## Introduction

Physical exercise has been considered as one of the cornerstones in the treatment of diabetes mellitus along with nutrition and medication since from the past 100 years ago [1]. Diabetes mellitus, a chronic metabolic disease, is characterized by an increase in the blood-glucose level resulting from a relative insulin deficiency or insulin resistance or both. As a consequence, it can lead to glycation of tissues, which proceeds with acute metabolic disturbances and ends with organ damage with severe health deteriorations. Research studies over the years, reported that the worldwide prevalence of diabetes mellitus appears to be increasing alarmingly. It is estimated that 5.4% of total population would be affected with the disease by the year 2025 as initial reports showed 4.0% in the year 1995. Thus, proper management should be done in order to treat diabetes mellitus and its complications [2]. 

Regarding the classifications of diabetes mellitus, two main categories have been elaborated i.e. type 1, insulin dependent diabetes mellitus, and type 2, non-insulin dependent diabetes mellitus. The present systematic review focuses on the type 2 diabetes mellitus (T2DM) with regard to its management for the simple reason that it is more common than type 1 diabetes mellitus. Interestingly, a total of 95% population presents with T2DM whereas only 5% are reported to have type 1 diabetes mellitus [3].

Regarding the management of T2DM, researches have highlighted the use of modern medicines, alternative or herbal medicines and exercise management therapy. The entire management regimen proves to have a positive impact on the disease. Nevertheless, the adverse effects of the medicines are also challenging, and it cannot be ignored. Therefore, physical activity or exercise is considered as the beneficial treatment regimen for the treatment T2DM [4,5]. To date, there are many reports on the role of physical exercise in managing T2DM. Throughout the world, many researchers have focused on the effect of physical exercise in T2DM with regard to its action, impact on laboratory parameters and organ damage either in the form of *in vivo* studies or clinical studies. Admittedly, there is lack of interest in practicing the exercise among the general population in the developing countries suffering from T2DM. There is paucity of studies in the Asian continent. The reason may be attributed to the lack of public awareness towards exercise in the world or even lack of evidence in highlighting the quantity with positive impact of exercises in T2DM. Therefore, main the aim of the present review was to summarize the findings from the published literature; focusing on the extent, type of exercise and its clinical significance in T2DM. The review mainly focused on the T2DM, regardless of obesity or metabolic syndrome. 

## Materials and Methods

Conforming to the usual patients, intervention, comparison and outcome (PICO) format, the following review was planned accordingly. Conforming to this format, our patients were those who were above ≥18 years; intervention being the exercise; comparison being the different types of exercises employed in the treatment of T2DM; and the outcome was the benefit of exercise in T2DM.

### Literature Search Strategy

The terms “type 2 diabetes mellitus”, “type 2 DM”, “NIDDM”, “exercise” and/or “physical activity” and “type 2 diabetes mellitus with exercise” were used to search the following databases: Pubmed, Science Direct, Scopus, ISI Web of Knowledge and Google scholar. The references of all saved articles were reviewed for relevant citations. 

### Inclusion Criteria

All publications or articles focused on human adults (≥18 years) in which there was information such as T2DM and exercise (types, modes and intensity of exercises), positive findings relating to the organs and biochemical parameters and studies based exclusively on exercise management, were included. All human or clinical cohorts, case-control studies, randomized controlled trials, randomized cross over studies, randomized cross pilot studies and pre-post design studies were included. Studies based on physical activity or exercise management in T2DM transcribed in English, between the year 2000 and 2012 were also included. In the case of similar studies with more than one publication, only the latest publication was taken for the present systemic review.

### Exclusion Criteria

The criteria such as articles written in other languages rather than English, case reports, case series, animal studies, letters to the editor and review articles were excluded. Moreover, the studies related to preventive measures of physical activity or exercises and studies with negative findings in T2DM were not selected. The justification for excluding preventive measurement of exercise in T2DM was because of the linkage of diabetes with obesity. In addition, most of the exercise associated preventive measurements come along with proper control of diet and with proper medication. In addition, the aim of present systemic review was to focus on positive effects of exercises towards T2DM [4,5]. In order to attain the uniformity of the studies, detailed and proper stringent criteria selections were employed in order to be included in this systematic review.

### Screening of Articles for Eligibility

Based on titles and abstracts, the articles were screened for eligibility. Two categories were made i.e. Category (1): included publications regarding the link between T2DM and exercise management therapy with potential outcomes, including laboratory parameters, organ changes or improvement and physical well-being. Category (2): indefinitely relating to the study design, sample size and/or aims towards the disease and its particular management. Regarding the articles falling under Category (1) and Category (2), only the full texts were obtained and assessed, based on the availability. Selection of articles was made by both authors and only those articles which were agreed upon by both, were eventually included in the present review. All included articles were read in detail and significant information was extracted.

### Data Extraction

From the selected publications, the following data such as type of study design; year of study; country of study; sample size; disease status; characteristic of exercises such as (type of exercise; duration of exercise; intensity of exercise); and findings/conclusions were extracted. In the case of any additional information needed, authors were contacted via email. The articles discarded in the present review were those in which the authors failed to be contacted. 

## Results

Over 4500 articles of exercises and T2DM-related articles were obtained with computer search. Majorities of these articles were systemic reviews, literature reviews and case reports. Articles published earlier than the year 2000, were not selected for our systemic review. A vast majority of articles were on the animal studies in exercise treated T2DM, which were eventually not included. Other reasons for excluding few articles were: (a) the study design not clearly mentioned (b) studies associated with T2DM and obesity or metabolic syndrome (c) studies associated with combined treatment of exercise and modern drug therapy, including oral hypoglycemic agents and insulin and (d) the articles which could not be retrieved in full text. 

Eventually, a total of 25 studies were eligible for the present systemic review. Majorities of the studies were designed as “Randomized Controlled Trial” (65%) and other study designs such as Cohort study, Case Control study, Pre-post design study, Quasi experimental design study were included under the remaining 35%. Majorities of exercise training programs based on aerobic exercises and a few studies were conducted on the resistance exercise. Few studies showed the combined therapeutic effect of aerobic and resistance exercises in T2DM. A large number of exercises treated diabetes researches were performed in the United States of America (USA). Following USA, there were other countries such as Australia, Italy, Brazil, Japan, Sweden, Ghana and Iran, which showed a positive therapeutic effect of exercise in T2DM. 

### Types of exercises in Diabetes mellitus

Diabetes mellitus is well known for having macro and micro vascular complications, which later proceeds to life-threatening conditions. Mortality as well as morbidity rate in diabetes mellitus is increasing, alarmingly. Effective management with less adverse effect is mandatory for managing the disease. Exercise training programs were alternative therapeutic regimens for both type 1 and T2DM. Especially, exercise management program influences T2DM more since it is an adult-onset disease and showed a promising effect on the community. Admittedly, the mechanism of the role of exercise on T2DM is not clearly understood. However, it can be determined that increase glucose uptake via glucose transporter 4 (GLUT4) to the skeletal muscle during the exercise is the responsible for reducing blood sugar level in T2DM patients (Figure S1). Exercise, including aerobic exercise, endurance type exercise, passive exercise and resistance exercise are fundamental therapeutic effects towardsT2DM.  

### Effect of aerobic exercise in T2DM

Aerobic exercise is the exercise which improves oxygen consumption and increases the functioning of the cardiovascular and respiratory systems. Aerobic exercise is a valuable therapeutic strategy for T2DM as it has beneficial effects on physiological parameters and reduces the metabolic risk factors in insulin resistance diabetes mellitus. Several studies have shown the positive effects of aerobic exercise based on different intensities on the improvement of T2DM. Aerobic exercises comprise of swimming, cycling, treadmill, walking, rowing, running and jumping rope [6,7]. Moderate aerobic exercise leads to maintenance of the blood pressure in diabetic neuropathy patients [8]. However, most of the randomized trial studies showed that high-volume aerobic exercise produced weight loss with significant improvement in insulin sensitivity [9]. Aerobic exercise improves the physiological parameters, including glycemic control, fasting blood-glucose level and lipid profile. Moreover, it can restore the endothelial function and reduces the arterial stiffness which is the positive denominator for developing cardiovascular complications in T2DM [10-12]. Both insulin and exercise increase glucose uptake into skeletal muscle via the glucose transporter (GLUT4) from an intracellular to the cell-surface [13,14]. In T2DM, there are deficiencies in the insulin receptors which result in impaired glucose uptake and GLUT4 translocation [15]. However, exercise therapy could restore the defects of insulin by providing GLTU4 translocation.

### Effect of resistance exercise in Type 2 DM

Resistance exercise leads to develop proper glucose control and less insulin resistance among T2DM. Resistance exercises are exercises that have to be performed against the resistance. Examples of resistance exercises include the weight lifting. Unlike aerobic exercise, resistance exercises are relied on the equipment. High and moderate intensities of resistance exercise range between 50-75% of 1-repetition maximum (1-RM)) [16]. A number of studies have documented the potential effects of aerobic training have been beneficial in the therapeutic regimen in T2DM patients. Similar to the aerobic exercise, resistance exercises are useful therapeutic tools in the management of T2DM. In addition, it is also proven to be safe and efficacious for the elderly insulin resistance diabetic patients. Resistance training has been reported to enhance insulin sensitivity, daily energy expenditure and quality of life [17]. Furthermore, resistance training has the potential for increasing muscle strength, lean muscle mass, and bone mineral density, which could enhance functional status and glycemic control and assist in the prevention of sarcopenia and osteoporosis [18,19]. 

### Other types of exercises in T2DM

Other types of exercise which act complementary regimes in chronic diseases like insulin resistance diabetes mellitus include endurance-type and passive exercise. Endurance exercise involves the use of several large groups of muscles, which depends on the delivery of oxygen to the muscles by the cardiovascular system. Passive exercise needs to include another person or outside force, or produced by voluntary effort of another segment of the patient's own body [16]. There are paucity of studies on these types of exercise in treating T2DM patients since a wide variety of studies supported the aerobic and resistance training program because of the beneficial effects. Few studies highlighted that endurance-type exercise also reduces postprandial hyperglycemia in T2DM patients. Yet, other uncommon types of exercises are the yoga classes and joba riding. To date, there have been conflicting reports on the yoga classes that have several positive impacts on T2DM. Some studies are successful to report that yoga classes training could improve the glycemic control in diabetic patients [20]. However, most of the researches could not show the statistical significance over these findings. Similar to that a randomized controlled trial proved that joba riding results in improving insulin sensitivity in T2DM patients [21]. In future, further studies with significant findings and detailed explanations are warranted to elaborate more on these types of exercise training programs. 

Past research findings related to types of exercise and intensity were tabulated [22-46] (Table 1). The table also depicted the study design adapted by various researchers all over the world and the salient findings. 

**Table 1:** Summary of selected studies.

**Year**	**Country**	**Sample size**	**Type of exercise**	**Intensity of exercise**	**Finding/conclusions**	**Study design**	**References**
2012	New Zealand	18 patients	Aerobic and resistance training	3 times/week, 16 weeks, 40-60 minute	Exercise has positive impacts on glycosylated haemoglobin (HbA1c), related diabetes markers (i.e. blood lipids, relevant cytokines and anthropometric and hemodynamic indices)	Cohort study	[22]
2012	Italy	25 patients	Aerobic and resistance training	3 times/week, 60 minutes, 4 months	Aerobic exercise reduces blood glucose concentrations to a greater extent than resistance exercise, and both have higher risk of exercise-induced hypoglycemia	Randomized controlled trial	[23]
2012	Italy	606 patients	Aerobic (treadmill, step, elliptical, arm or cycle-ergometer) and resistance training	2 times/week, 12 months,55% - 70% of predicted maximal oxygen consumption (VO2max) for aerobic exercise,60% of predicted 1-Repetition Maximum (1-RM) for resistance exercise	Low intensity exercise is as effective as high intensity exercise in reducing risk factors for cardiovascular disease in T2DM	Multicenter randomized controlled trial	[24]
2012	Netherlands	40 patients	Resistance exercise and endurance type exercise	24hr period, 45 minutes session, resistance type exercise (75% one repetition maximum) and endurance-type exercise (50% one maximum workload capacity)	Both resistance- and endurance-type exercise can be integrated in exercise intervention programmes designed to improve glycaemic control.	Randomized crossover study	[25]
2011	Netherlands	20 patients	Aerobic or resistance training	12 weeks	Exercise improves blood glucose regulation (HbA_1_c), muscle strength (isometric peak torque)	Pre-post design study	[26]
2011	Australia	34 patients	Cardiorespiratory and resistance exercise	4 weeks, 2 session/week, (1 hr supervised and 30 minutes unsupervised)	Decrease in blood glucose, resting heart rate, systolic blood pressure and increase in cardiorespiratory fitness with short-term exercise training	Quasi experimental design	[27]
2011	Brazil	10 patients	Resistance and aerobic exercise	24 hr period, 60 minutes interval	Single bout of resistance exercise decreases blood pressure in T2D patients over a 24h period, more effective than aerobic exercise	Randomized controlled trial	[28]
2011	Ghana	18 patients	Prescribed aerobic exercise	3 times/week, 30 minutes, 50-75% maximum heart rate	Aerobic exercise improves physiological parameters such as fasting blood glucose level and lipid profile level in T2DM patients	Randomized controlled trial	[29]
2010	Netherlands	9 patients	Isoenergetic bout of endurance - type exercise	Low-intensity, 60-30 minutes, 24 hrs	Single bout of low- intensity exercise reduces post prandial hyperglycemia	Randomized cross over study	[30]
2010	Iran	65 patients	Aerobic exercise	16 weeks (3 days/week, 90 min, 50-80%VO2max)	Aerobic exercise show potential reduction of glycosylated hemoglobin values in T2DM patients	Randomized controlled trial	[31]
2010	United States of America, Los Angeles	262 patients	Aerobic and resistance training	150 minutes/ week, 9 months, 50% to 80% of maximum oxygen consumption.	Combination of aerobic and resistance training improved HbA(1c) levels	Randomized controlled trial	[32]
2010	Japan	24 patients	Joba riding	7 times/ week, 30 minutes, 3 months	Daily Joba exercise is potentially useful in improving insulin sensitivity and resting metabolism in T2DM patients	Randomized controlled trial	[33]
2010	Singapore	68 patients	Progressive resistance exercise and aerobic exercise	2 times/day, 50 minutes, for 8 weeks	Progressive resistance exercise has similar effects to aerobic exercise towards T2DM patients	Randomized controlled trial	[34]
2009	Brazil	40 patients	Physical (treadmill) exercise	3-5 times/ week, 30 minutes walks, for 20 weeks at 70% maximum heart rate	High frequency of regular exercise showed significant effect on glycemic control in T2DM	Cohort study	[35]
2009	London	59 subjects	Yoga classes	2 times/week, 90 minutes, 12 weeks	Yoga (exercises) reduced HBA1C level in T2DM patients (statistically not significant)	Exploratory randomized controlled trial	[36]
2006	United States of America	30 patients	Resistance training	16 weeks	Resistance training results in muscle hypertrophy and improves glycemic control in patients with type 2 diabetes.	Randomized controlled trial	[37]
2006	United States of America	62 patients	Strength training exercise	16 weeks	Strength training exercise improved muscle quality insulin sensitivity and metabolic control	Randomized controlled trial	[38]
2004	Japan	40 patients	Aerobic exercise (bicycle ergometer)	40 minutes/day, 5 days, at 3 weeks intervention	Aerobic exercise restore the insulin sensitivity with regardless of changes in adiponectin	Randomized cross over study	[39]
2004	Australia	13 subjects	Short-term exercise training	3 days/week, 120 minutes, 8 weeks	Short-term exercise enhances insulin sensitivity and reduces triglyceride level in T2DM patients compared to control subjects	Case control study	[40]
2003	Finland	50 patients	Resistance training exercise	30 minutes/day, for 12 months, 10-12 times repetitions	Resistance training exercise program improves the baroreflex modulation of cardiovascular function which can result in as preventive measures for sudden cardiac death in T2DM patients	Randomized controlled trial	[41]
2003	United States of America, Boston	75 patients	Walking exercise	3 times/ week, 60 minutes walking, for 12 weeks	Simple exercise improves glycemia and cardiovascular risk factors in T2DM subjects	Randomized controlled pilot study	[42]
2002	United States of America	62 patients	Resistance training exercise	3 times/week, 45 minutes, 16 weeks	Exercise showed positive effect towards glycemic control and metabolic outcomes in T2DM	Randomized controlled trial	[43]
2001	Australia	16 patients	Exercise (Bicycle ergometer, treadmill walking, resistance training)	1 hr/day, 70%-80% of Heart rate for bicycle and walking, 55%-65% for resistance training	Combined aerobic and resistance exercise restore endothelial dysfunction in patients with vascular disease occurred in T2DM	Randomized cross over study	[44]
2001	Sweden	15 subjects	Exercise (cycle ergometer)	45 minutes/day, 70% of workload, acute exercise	Normal exercise improves AMPK activity which is an attractive target for the treatment of T2DM	Case-control study	[45]
2001	Japan	50 patients	Walking and cycling exercise	5 times/week, 1 hr, 50% maximum oxygen uptake	Exercise training in T2DM subjects reduces serum leptin levels	Randomized controlled trial	[46]

## Discussion

Diabetes mellitus is a chronic endocrine disorder, and it needs the definite treatment. Several complications are associated with diabetes, and with lack of proper treatment would result in life-threatening condition. Many researches have shown that exercise plays a crucial role in improving T2DM. Exercise not only improves the glycemic control, but it can also improve the insulin sensitivity and restore the diabetic associated complication such as cardiovascular damage, which considered as one of the major complications. Based on the past findings, the present systemic review summarized the extent and the type of exercise among the T2DM population. 

From our systemic review, it was revealed that compared to aerobic exercise, resistance exercise could create fewer impression on the diabetic patients as few studies were found to use resistance exercise as an alternative therapeutic agent in T2DM. Primarily, it can be due to the fact that resistance exercises depend on the use of equipment. It can be costly effective and needs the proper supervision. For the sedentary people to undergo the resistance training, it has the high risk in discontinuation of the training program due to its negative aspect [47]. On the other hand, aerobic exercise comprises of simple training programs which are devoid of using equipment and it showed several positive impacts on T2DM [10-13]. It is also agreed upon by the other researchers who highlighted that many aspects are concerned towards resistance exercise such as knowledge of exercise, economic aspect of exercise, sustainability of the exercise [48]. However, both trainings have synergestic effects on the insulin sensitivity [48]. 

In the present systemic review, it was also revealed that most of the training programs were carried out approximately three times in a week. The advantage being that it would be easier to schedule fewer and longer sessions rather than frequent and shorter sessions for the patients. For aerobic exercise, increase in insulin sensitivity level was observed with single bouts of exercise. Perhaps, it depends on the duration and intensity of the exercise [49]. As discussed earlier, increase insulin sensitivity normally lasts not more than 72 hours, and it can be concluded that regular exercise or physical activity three times/week results in definite and effective management for T2DM patients [50]. The frequency, duration, total time and intensity rating of different studies on exercise [51-56] were also highlighted (Table 2).

**Table 2:** Table showing the descriptive statistics regarding exercise in different races.

**Ethnicity**	**Frequency per week**	**Duration in weeks**	**Total time in hours**	**Intensity rating**	**References**
African American	3	13	19.5	3	52
European	3	8	18	5	53
Japanese	7	7	14	1	54
Hispanic	3	16	36	4	55
Polynesian, European	3	10	30	3	56

It cannot be firmly stated that the treatment of diabetes with exercise showed promising role only in USA but there are many interesting facts to ponder. The number of studies performed in USA is more than in any other part of the world and this raises speculations on the awareness of exercise treatment in T2DM in USA. It may also be stated that the strategy of distribution the knowledge of exercise in the population is highly effective. This may be the possible reason why the incidence of diabetes is decreasing surprisingly in the USA population. An important reason is that many of the clinical related researches depend on the cooperation from the patients and their consent. Hence, it is important to share the proper information about the advantages of exercise in diabetes in order to perform a clinical study. 

It was reported that exercise has a positive role in maintaining the glycemic level, increasing the insulin sensitivity and also improving cardiovascular risk factors with regard to T2DM [32,37,38,42,43]. Exercise causes prolonged glucose homeostasis and continuous glucose monitoring (CGM) technology is important to assess exercise associated hypoglycemia [57]. Aerobic and anaerobic exercise leads to decrease in blood glucose and increase in blood glucose, respectively in individuals with type 1 diabetes mellitus [57]. It is reported that exercise associated hypoglycemia is also a major hurdle for exercise participation in adults and younger individuals and CGM may prove to be effective in sportspersons [58,59]. Appropriate carbohydrate and insulin modifications during exercise can be effectively done by CGM. In individuals withT2DM, the CGM technology has been reported to be a useful adjunct to exercise counseling and lifestyle intervention [57]. 

Some of the adverse effects of exercises for patients with T2DM need to be discussed in detail. Although exercises are effective among these patients, the risk of sudden cardiac death has been suggested among diabetes patients who have coronary heart disease [60]. Therefore, it is recommended that prior screening for myocardial ischemia is necessary before any exercise prescription for patients with diabetes [60]. Failure in heart rate recovery post exercise was reported among diabetes patients as a result of cardiac autonomic neuropathy [61]. Hence, clinicians may consider finishing the exercise session with a cool down exercise session. In addition to it, the vital sign need to be monitored following cessation of exercises. Diabetic adults with complications such as retinopathy, nephropathy and neuropathy may respond with lesser acceptability to exercises compared to diabetic patients without any additional complications [62]. In such cases, exercise professionals may provide additional support in exercises or even design tailor made exercises to individual diabetic patients. Furthermore, post exercise phosphocreatine recovery and mitochondrial oxygenation was reported to be impaired among diabetes patients with lower extremity complications [63]. Taking this into account, the nature of exercises should be restructured for patients accordingly. Perhaps, endurance exercises such as swimming which does not involve gravitational stress on lower extremities may be considered instead of land-based aerobic exercise program. Slow oxygen uptake which results in exercise intolerance is another factor to be considered during sub-maximal exercises [64]. 

Evidence supports that the skeletal muscles of T2DM patients demonstrate imbalance in oxygen delivery to muscles compared to the oxygen intake [64]. This may result in post exercise fatigue and tiredness. Late onset of hypoglycemia following aerobic exercises is also another risk. Hence, clinicians may watch out and educate patients for hypoglycemic symptoms during their exercise training [23]. In summary, we would like to propose that practitioners may take account of the above said adverse effects of exercises before exercise prescription to patients with diabetes.

One of the limitations of the study is that the present review did not emphasize on the statistical analysis since many of the earlier reviews already focused on the statistical aspects of published literature. Admittedly, another limitation may be the terminologies related to the English language, full text retrieval and Thesaurus differences in different database. Confining the search to selected databases may be another limitation.

## Conclusion

The present review was planned to reveal the importance of types of exercise and prevalence of exercise management in T2DM across the world, within recent years. It is expected that this systemic review would attract many researchers across the world especially in developing countries to perform further studies based on exercise management in diabetes or T2DM. From the published data, it can be also concluded that exercise based research for diabetes are less in the Asian countries. This may be attributed to the strong perception of medication for the disease in Asians. Yet another, medication comprises of major or minor side effects for the disease. It is noteworthy that the effect of exercise revealed beneficial results for T2DM deprived of any untoward effect. The present review showed that exercise related studies in diabetes were performed only in few selected countries in Asia such as Singapore, Iran and Japan and this justifies the utmost importance of future studies in Asian population. Detailed researches and further studies with certain distribution of information related to the importance of exercise in T2DM may be essential for the population in the developing countries. 

## Supporting Information

Figure S1 **Schematic diagram showing role of exercise in type 2 diabetes mellitus.**
(TIF)
